# Haemorrhagic Complications After Microsurgical Treatment for Intracranial Aneurysms Under Acetylsalicylic Acid: An Impact Analysis

**DOI:** 10.7759/cureus.62622

**Published:** 2024-06-18

**Authors:** Anton Konovalov, Fyodor Grebenev, Anton Artemyev, Vadim Gadzhiagaev, Yuri Pilipenko, Dmitry Okishev, Alina Manushkova, Shalva Eliava, Bipin Chaurasia

**Affiliations:** 1 Cerebrovascular Surgery, Burdenko National Medical Research Center of Neurosurgery, Moscow, RUS; 2 Neurosurgery, Burdenko National Medical Scientific Research Centre of Neurosurgery, Moscow, RUS; 3 Neurosurgery, Educational Institution of Higher Education Sechenov First Moscow State Medical University, Moscow, RUS; 4 Neurosurgery, M.F. Vladimirsky Moscow Regional Scientific Research Clinical Institute, Moscow, RUS; 5 Vascular Surgery, Burdenko National Medical Research Center of Neurosurgery, Moscow, RUS; 6 Anesthesiology, Burdenko National Medical Research Center of Neurosurgery, Moscow, RUS; 7 Neurosurgery, Bhawani Hospital and Research Centre, Birgunj, NPL

**Keywords:** pterional craniotomy, microsurgical aneurysm clipping, aneurysm, hemorrhage, acetylsalicylic acid

## Abstract

Background: Patients with intracranial aneurysms often have comorbidities that require them to take acetylsalicylic acid (ASA). In recent years, many patients with aneurysms have been prescribed ASA to prevent aneurysm enlargement. ASA is also prescribed to patients with intracranial aneurysms in preparation for surgical revascularization.

Methods: From 2016 to 2021, 64 patients underwent microsurgical aneurysm clipping without revascularization, and an additional 20 patients underwent extracranial to intracranial (EC-IC) bypass. The following parameters were analysed: the frequency of hemorrhagic complications, the blood loss volume, the duration of surgery and inpatient treatment, the change in hemoglobin level (Hb), hematocrit (Ht), erythrocytes, and clinical outcomes according to the modified Rankin scale (mRS).

Results: At the time of surgery, laboratory-confirmed effect of the ASA was registered in 22 patients (main group). In 42 patients, the ASA was not functional on assay (control group). Hemorrhagic complications were noted in two patients in the ASA group. In both cases, the hemorrhagic component did not exceed 15 ml in volume and did not require additional surgical interventions. Statistical analysis showed no significant differences in hemorrhagic postoperative complications.

Conclusion: Taking low doses of acetylsalicylic acid during planned microsurgical clipping of cerebral aneurysms does not affect intraoperative blood loss volume, risk of postoperative hemorrhagic complications, length of stay in the hospital, or functional outcomes.

## Introduction

Acetylsalicylic acid (ASA) is the most widely used antiplatelet drug for the prevention of cardiovascular diseases [1]. ASA inhibits platelet aggregation, which allows the use of this drug for the prevention of myocardial infarction, ischemic stroke, and thrombotic complications after open vascular interventions [2]. Because of the risk of intra- and postoperative hemorrhagic complications, many surgeons cancel ASA therapy more than seven days before elective neurosurgical interventions [3]. Some studies have demonstrated the effect of ASA on the risk of bleeding during surgical interventions [4,5], while in other studies, perioperative discontinuation of aspirin increased the risk of cardiovascular disease [6,7]. Very few studies have been devoted to the use of the ASA and planned neurosurgical interventions [3,8-10]. It has been proven that ASA affects platelet function in only 60-80% of cases [11]. The effect of ASA on platelet function can be verified by various laboratory tests. Most studies evaluating perioperative risks in patients receiving ASA have not performed a confirmatory test.

While many studies show high hemorrhagic risks in neurosurgical patients admitting ASA, it has been reported that the use of ASA in patients undergoing emergency neurosurgical interventions for ruptured aneurysms did not affect intraoperative blood loss or the risk of hemorrhagic complications [8]. Patients with intracranial aneurysms often have comorbidities that require them to take an ASA. In recent years, many patients with aneurysms have been prescribed ASA to prevent aneurysm enlargement [12,13]. Additionally, in accordance with the protocol adopted in many clinics, ASA is prescribed to patients with intracranial aneurysms in preparation for surgery using revascularization [14].

The purpose of this study is to evaluate the laboratory-confirmed effect of ASA on the risk of blood loss and hemorrhagic complications in the planned microsurgical treatment of cerebral aneurysms, as well as to assess the frequency of hemorrhagic complications during revascularization operations while taking ASA in patients with aneurysms.

## Materials and methods

All patients included in this study underwent microsurgical treatment at our center from 2016 to 2021 for an intracranial aneurysm. Inclusion criteria for the study were as follows: 1) either the patient had an unruptured aneurysm or more than 21 days had passed from rupture to the moment of the treatment; 2) microsurgical intervention for an aneurysm; 3) available results of analysis of the effect of ASA; and 4) availability of CT data before and after surgery. The exclusion criteria were 1) treatment performed within 21 days after the rupture (an acute period of subarachnoid hemorrhage (SAH)); 2) the absence of data on platelet function; and 3) the presence of conditions not associated with neurosurgical pathology that affected the risk of complications or the duration of treatment (for example, pulmonary embolism, acute myocardial infarction, acute kidney injury, etc.

Analysis of the functioning of ASA was carried out using inducer substances (collagen/adenosine diphosphate (ADP), collagen/epinephrine), or the functioning of the platelet P2Y12 receptor was evaluated. The test was considered positive in the case of the following values: 1) >121.0 sec for the ADP test, 2) >160.0 sec for the epinephrine test, and 3) >106.0 sec for the P2Y12 test.

The study was divided into two parts. In the first part (retrospective cohort study), all patients who underwent the ASA effect test and microsurgical aneurysm clipping were collected. The main group of patients was formed from patients who had a laboratory-confirmed effect of ASA, and the control group consisted of patients without the effect of ASA (the assay showed no effect). Comparison of the two groups was carried out according to the following parameters: the frequency of hemorrhagic complications, the blood loss volume, the duration of surgery and inpatient treatment, the dynamics of hemoglobin (Hb), hematocrit (Ht), erythrocytes, and clinical outcomes according to the modified Rankin scale (mRS).

The blood loss volume was assessed according to medical records, operation protocols, and anesthesia cards. The volume of blood loss was compared with the dynamics of hemoglobin (Hb), hematocrit (Ht), and erythrocytes during hospitalization.

In the second part, the frequency of intraoperative and postoperative hemorrhagic complications was assessed in patients with revascularization procedures over the specified period. All patients in this group received ASA before surgery. Patients in this group were not included in the original comparison groups, since all, without exception, received aspirin before surgery intentionally to reduce the risk of anastomotic thrombosis. Additionally, the complexity of interventions due to the complex anatomy of the aneurysm was incomparably higher in comparison with operations of standard clipping. Given these factors, it was inappropriate to compare patients in the revascularization group with those in the simple clipping group. Additionally, as in the first part of the study, the frequency of complications, blood test parameters before and after surgery, the volume of blood loss, the duration of surgery and inpatient treatment were evaluated.

Statistical data analysis was performed using SPSS® software Version 23.0 (IBM Corp., Armonk, NY). Tables and graphs were constructed using SPSS® Statistics Version 23.0 and Microsoft® Excel for Mac Version 16.67. The normality of the sample distribution was determined using the Kolmogorov-Smirnov test. The determination of the presence of a statistically significant difference was carried out using Pearson's Chi-square test if the studied parameter was qualitative. If more than 20% of the cells had frequency values less than 5 when calculating Pearson's Chi-square, the Yates correction was applied. In addition, the likelihood ratio and Fisher's exact test were used. If the studied parameter was quantitative, then the assessment of the statistically significant difference was made using analysis of variance (ANOVA) provided the parameter was normally distributed. Otherwise, the Mann‒Whitney U test was used for two independent samples.

## Results

Influence of ASA in standard microsurgical clipping

From 2016 to 2021, 3434 patients with intracranial aneurysms underwent simple clipping without revascularization in our center. Sixty-four of them were on ASA perioperatively and had assay performed. The simple clipping group included 25 males (39.1%) and 39 females (60.9%). The mean age of the patients was 57±9 years (range 29-70 years).

At the time of surgery, the laboratory-confirmed effect of the ASA was registered in 22 patients (main group), and in 42 patients, the ASA did not function (control group) according to the assay. The values of the assessed parameters mentioned earlier are shown in Table 1. Statistical analysis showed no significant differences in hemorrhagic postoperative complications (Yates-adjusted Chi-square test = 0.341, p = 0.559, likelihood ratio = 1.363, p = 0.243, p-value for Fischer's exact test = 0.270) (Table 1).

**Table 1:** Comparison of the main and control groups (the first part of the study) Hb – hemoglobin level; Ht – hematocrit level; RBC – red blood cell count.

Parameter	Antiplatelet group (n=22)	Non-antiplatelet group (n=42)	P-value
Hemorrhagic complications	2 (9.1%)	1 (2.4%)	0.559
Inpatient treatment (days)	245.91 ± 211.1	184.54 ± 84.5	0.284
Blood loss volume (ml)	266.59 ± 96.8	254.93 ± 67.7	0.788
Duration of the procedure (min)	12.05 ± 5.4	10.76 ± 3.7	0.545
Hb pre-op (g/L)	129.41 ± 15.9	134.07 ± 14.3	0.237
Hb post-op (g/L)	109.55 ± 14.5	120.50 ± 14.7	0.006
Hb decrease after operation (%)	-15.18 ± 7.0	-9.99 ± 7.5	0.009
Ht pre-op (%)	39.02 ± 4.3	40.02 ± 4.2	0.373
Ht post-op (%)	32.83 ± 4.6	35.85 ± 4.3	0.011
Ht decrease after operation (%)	-15.81 ± 8.3	-10.23 ± 7.8	0.01
RBC pre-op (10^12^/л)	4.53 ± 0.5	4.51 ± 0.5	0.902
RBC post-op (10^12^/л)	3.75 ± 0.5	4.01 ± 0.5	0.039
RBC decrease after operation (%)	-17.12 ± 7.8	-10.90 ± 7.6	0.003
mRS postoperatively			
mRS=0	18	31	1.0
mRS=2	2	5	
mRS=3	0	2	
mRS=4	1	2	
mRS=6	1	0	

**Figure 1:**
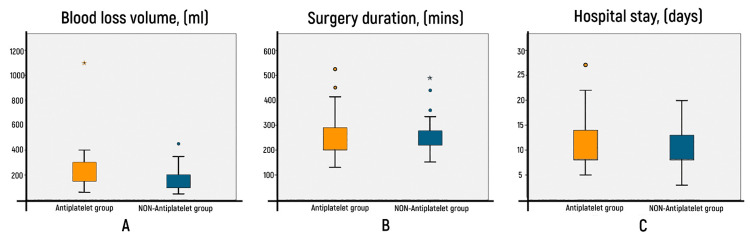
Graphical comparison of the characteristics of both groups A - blood loss volume (ml), B - duration of the operation (minutes), C - duration of inpatient treatment (days).

The average blood loss volume in the main group was 245.91 ± 211.1 ml (range 60-1100 ml), and in the absence of the ASA effect, it was 184.52 ml ± 84.5 (range 50-450 ml) (Figure 1). These differences were not statistically significant (Mann-Whitney U = 388.000, p = 0.284) (Figure 1A). The distribution of mean values of hemoglobin, hematocrit, and erythrocytes before and after surgery and statistical analysis of differences are presented in Table 1. Initially, these parameters did not differ significantly between the two groups.

The mean hemoglobin level before surgery in the ASA group was 129.41 ± 15.9 g/L. On average, the decrease in hemoglobin level was 15.2% (up to 109.55 ± 14.5 g/l) in the patients with ASA effect compared to 10% (9.99% ± 7.5) in the group without ASA. The mean red blood cell count before surgery in the ASA group was 4.53 ± 0.5*1012/l, with a decrease of 17.1% postoperatively. In the group without ASA, the decrease comprised only 10.9%. The hematocrit in the ASA group averaged 39.02 ± 4.3%. In the group without ASA, the average hematocrit before surgery was 40.02 ± 4.2%. Statistical differences after surgery were significant between the two groups in all three parameters.

The average duration of the operation in the case of the ASA effect was 266.59 ± 96.8 minutes, and in the absence of the ASA effect, it was 254.93 ± 67.7 minutes (Figure 1). These differences were not statistically significant (p = 0.788). The average number of bed days in the case of ASA functioning at the time of surgery was 12.05 ±5.5 days (range 5 - 27), and in the absence of ASA functioning, it was 10.76 ±3.7 days (range 3 - 20). These differences were also not statistically significant (Mann-Whitney U = 419.50, p = 0.545).

Next, we conducted an analysis of the functional status of the patient according to the modified Rankin scale after the operation, which we managed to carry out for 62 patients. The obtained differences in the groups were not statistically significant (Pearson's Chi-square test = 3.104, p = 0.541; likelihood ratio = 4.016 p = 0.404).

**Figure 2:**
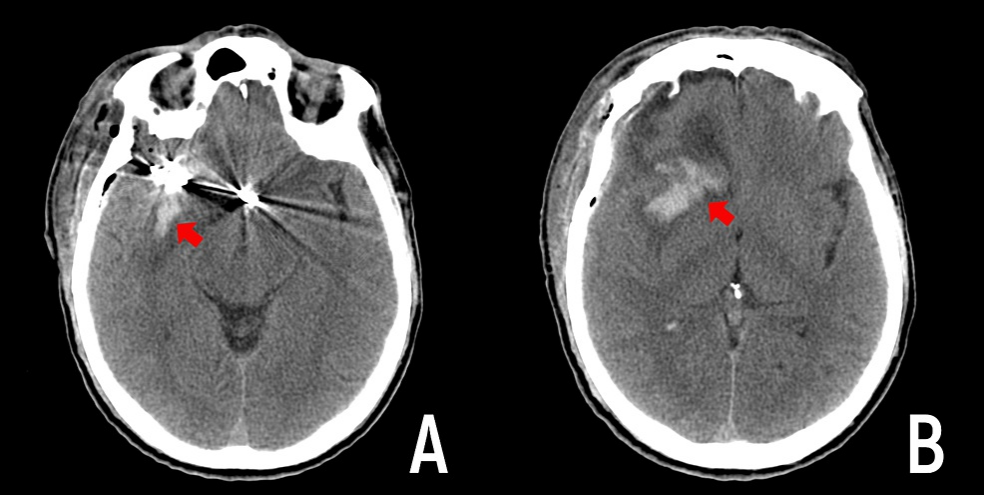
Hemorrhagic complication in the first case CT scan of the head on the first day after clipping of the aneurysms of the right middle cerebral artery and anterior communicating artery. A - Hemorrhagic imbibition is located along the Sylvian fissure with the transition to the right frontal lobe (red arrow). B - Hemorrhagic imbibition of the deep portions of the right frontal lobe with perifocal edema (red arrow).

**Figure 3:**
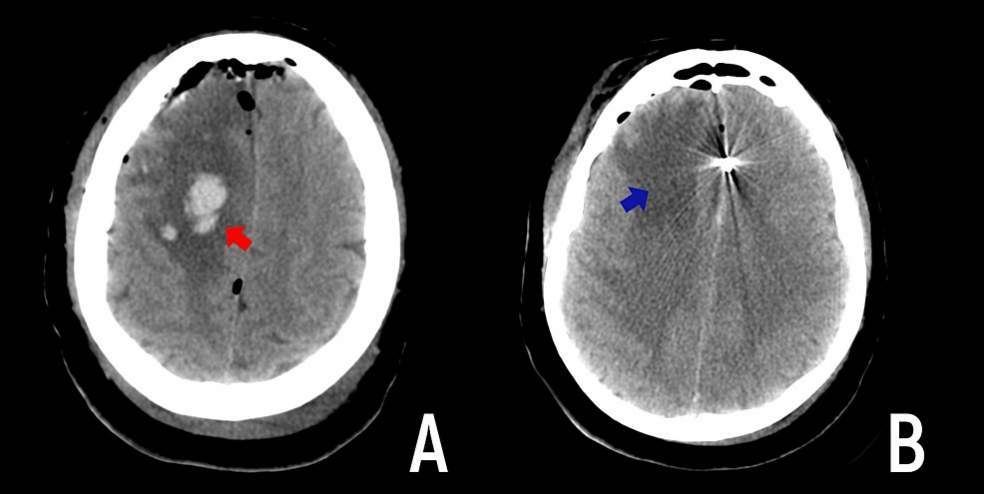
Clinical observation-hemorrhagic complication in case number 2 CT scan of the head on the first day after clipping of the aneurysm of the right pericallosal artery through the interhemispheric approach. A pronounced swelling of the right frontal lobe with hemorrhagic impregnation (red arrow) is shown. B - Venous ischemia of the right frontal lobe (blue arrow).

Hemorrhagic complications were noted in two patients in the ASA group (Figures 2, 3). In both cases, the hemorrhagic component did not exceed 15 ml in volume and did not require additional surgical interventions. In the first case, there was no clinically significant neurological deficit in the patient. In the second case, the patient had a focal neurological deficit that had partially regressed by the time of discharge.

Influence of ASA during revascularization operations

This group included 20 patients who underwent extracranial to intracranial (EC-IC) bypass: five men (25%) and 15 women (75%) (Table 2). ASA functioned in 17 cases (85%) and did not function in three cases (15%). In our cohort, we identified four Posterior Inferior Cerebellar Artery (PICA) aneurysms, 11 Middle Cerebral Artery (MCA) aneurysms, and five intracranial carotid artery (ICA) aneurysms requiring a bypass procedure. For patients with PICA aneurysms, an Occipital Artery (OA)-PICA EC-IC bypass was performed. In those with ICA and MCA aneurysms, either single-barrel or double-barrel superficial temporal artery-middle cerebral artery (STA-MCA) EC-IC bypasses were utilized. All aneurysms in this subgroup were characterized as complex-being, wide-necked, large or giant, atherosclerotic, or thrombotic.

**Table 2:** Microsurgical revascularization group (mean values) Abbreviations: Hb – hemoglobin level; Ht – hematocrit level; RBC – red blood cell count.

Parameter	Value (mean ± standard deviation)
Total number of patients	20
Age (years)	45.05 ± 11.6
Hemorrhagic complications	1
Inpatient treatment (days)	17.10 ± 10.3
Blood loss volume (ml)	425.00 ± 252.1
Duration of the procedure (min)	571.00 ± 149.5
Hb pre-op (g/L)	132.80 ± 14.9
Hb post-op (g/L)	105.00 ± 17.0
Hb decrease after operation (%)	-20.895 ± 9.1
Ht pre-op (%)	39.73 ± 4.0
Ht post-op (%)	31.16 ± 4.9
Ht decrease after operation (%)	-21.5335 ± 9.3
RBC pre-op (10^12^/л)	4.5995 ± 0.4
RBC post-op (10^12^/л)	3.5485 ± 0.5
RBC decrease after operation (%)	-22.805 ± 9.2
mRS postoperatively	
mRS=0	13
mRS=2	1
mRS=3	5
mRS=5	1

**Figure 4:**
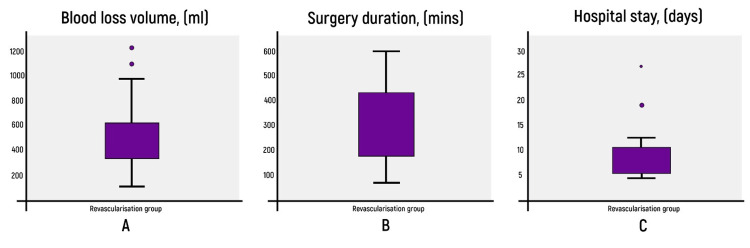
Group of patients after microsurgical revascularization A - blood loss volume (ml); B - duration of surgical intervention (minutes); C - duration of hospital stay (days).

The collagen/epinephrine test was performed in all 20 patients. Collagen/ADP was additionally investigated in one patient, P2Y12 was additionally studied in four patients, and both collagen/ADP and P2Y12 were additionally studied in one patient. The study revealed the only clinically insignificant hemorrhagic complication, which comprised 5% of the total number of patients. The average blood loss volume in this group was 425.00 ± 252.1 ml. The duration of the operation averaged 571 ±149.5 minutes. The inpatient treatment averaged 17.1 days ±10.3 (Figure 4).

## Discussion

ASA irreversibly blocks the enzyme system of platelet cyclooxygenase, preventing the formation of thromboxane A2 and inhibiting platelet aggregation for the entire lifetime of the affected platelet (approximately 10 days) [15]. This block occurs even at the lowest commonly prescribed therapeutic and prophylactic dose of ASA, 100 mg per day [15]. Based on the normal rate of platelet production, it takes approximately five to six days after discontinuation of ASA to replenish approximately 50% of circulating platelets (10% every 24 hours) [16]. ASA does not cause a general disturbance of the hemostasis system unless it is prescribed to patients with concomitant hemostasis disorders such as hemophilia, uremia, or anticoagulant therapy-induced coagulopathy [16].

In the context of aspirin administration in patients with intracranial aneurysms (IAs), existing evidence suggests that inflammation plays a critical role in the structural change of the aneurysm wall and its subsequent rupture [17]. Several observational studies have linked the use of nonsteroidal anti-inflammatory drugs (NSAIDs) with a decrease in the growth rate of IA and a lower risk of developing Aneurysmal Subarachnoid Hemorrhage (aSAH) [18-20]. Both animal experiments and human clinical studies have shown that vascular remodeling and inflammatory cascades play a critical role in the formation, progression, and rupture of IA [21]. Abnormal wall shear stress activates the prostaglandin (PG)E2-EP2 pathway in endothelial cells (ECs) at an early stage of intracranial aneurysm formation [22]. Subsequently, apoptosis and migration of vascular smooth muscle cells, accompanied by inflammatory cell infiltration, lead to degradation of the vascular wall, which in turn leads to the progression and rupture of IA [21]. Hasan et al. demonstrated in a small group of patients who cyclooxygenase-2 (COX2) and microsomal prostaglandin synthase E2-1 (mPGES-1) are expressed in human intracranial aneurysms, and their expression increases with aneurysm rupture, which can be blocked by ASA [23]. ASA is widely prescribed as a standard prophylaxis for patients at risk of cardiovascular and cerebrovascular diseases [16]. In this regard, in recent years, it has often been recommended to continue ASA therapy in the perioperative period, including during operations on intracranial aneurysms. We tried to assess the true impact of ASA on the risk of complications and assess whether it is worth canceling low doses of ASA due to the risk of hemorrhagic complications.

In a study by Nakamizo et al., the incidence of hemorrhagic complications among patients operated on for IAs was higher when taking antithrombotic therapy [9]. At the same time, a study by Toyoda et al. found that neither dual antiplatelet therapy nor their combination with warfarin increased the risk of perioperative bleeding [24]. According to our data, the use of ASA did not lead to an increased risk of hemorrhagic complications in the microsurgical clipping group and was not associated with a high frequency of hemorrhagic complications in the revascularization group. Hanalioglu et al. achieved similar results in their work. In their study, perioperative use of ASA was not associated with an increased incidence of hemorrhagic complications after the removal of intracranial tumors [25]. As mentioned earlier, ASA is often used in preparing a patient for revascularization surgery in many clinics around the world. According to a number of studies, the frequency of hemorrhagic complications did not increase, despite the increased duration of the operation [10].

When assessing the risk of bleeding during surgery, the groups with ASA and without ASA did not differ statistically in our study. However, when assessing blood parameters (erythrocytes, Hb, Ht), there were significant differences in the group with and without ASA. We attribute this not to the effect of ASA but to the hemodilution after the infusion load after surgery, since there were no significant differences between the groups in terms of blood loss. If we compare absolute and relative values, then the changes are not clinically significant. There was no significant effect on functional outcome or risk of hemorrhagic complications. However, there were indeed two patients with hemorrhagic complications in the ASA group. We did not find a significant difference, which may indicate that these complications may be associated with venous congestion and ischemia. There were also no significant differences in clinical outcomes. In our opinion, cases of hemorrhagic complications are not associated with the direct use of an ASA. This is supported by data from a series of 20 patients with microsurgical revascularization. ASA also functioned in this group, and despite the increased duration of the operation, there was only one hemorrhagic complication. We noted that in this group, the blood loss volume was greater than that in the simple clipping group, which is associated with an increased duration of surgical intervention.

Anesthesiology and cardiology guidelines recommend the perioperative use of ASA in patients with low to moderate bleeding risk and high cardiovascular risk [26]. Intracranial surgery, as well as spine and spinal cord surgery, are considered procedures with a high risk of bleeding and are therefore excluded from recommendations for continuing antiplatelet agents even in patients with high cardiovascular risk [27]. A national survey of neurosurgeons in Germany studying the use of ASA before intracranial surgery showed that 77.5% of respondents believe that patients taking low doses of ASA have an increased risk of major perioperative hemorrhage [3]. Of these respondents, 58% reported personal experience of having patients with perioperative bleeding [3]. It should be noted that in studies that do not have data on the functional state of platelets and the confirmed effect of ASA, the evidence for such statements is limited. As our study showed, taking an ASA does not mean that there is an actual effect on platelet function. Thus, in patients in the microsurgical revascularization group, ASA had no effect in 15% of cases. According to the literature data, resistance to ASA is present in 5-45% of cases [11]. There are two types of assays assessing the ASA activity: the test for induced and spontaneous platelet aggregation. The first is carried out with the connection of substances-inductors, and the second is carried out without auxiliary activators [16]. An analysis of platelet function is carried out using universal aggregation inducers (UIA) - components similar in chemical composition to compounds that are present in human vessels and activate the thrombus formation process - ADP, collagen, epinephrine. The aggregation activity of platelets is determined by the difference between the light density of the blood before the start of the thickening process and after reaching the maximum aggregation using an aggregometer. Additionally, one of the options for assessing platelet function is an analysis of the functioning of the P2Y12 receptor. Therefore, all patients taking ASA need to assess the functional state of platelets using available methods.

Study limitations

The main disadvantage of the study is its retrospective nature. In this regard, there is a risk of errors in the collection of pre and postoperative data. The second part of the study was performed without the use of the control group, and therefore, the data obtained are only observational in nature; however, they emphasize the significance of the data obtained from a comparative analysis of two groups of simple clipping. Nevertheless, it is worth noting that the study is based on a relatively large group of patients with a rare pathology, and statistical methods of analysis were used that meet modern standards; therefore, the results of the work can serve as an important addition to existing knowledge on the given problem.

To obtain more reliable data and develop clinical recommendations in preparing a patient for a neurosurgical operation, it is necessary to conduct multicenter randomized trials with an assessment of the effect of ASA in neurosurgical interventions.

## Conclusions

Administering low doses of acetylsalicylic acid prior to microsurgical clipping of cerebral aneurysms appears to have no significant impact on the volume of blood lost during surgery. Additionally, it does not seem to increase the likelihood of hemorrhagic complications post-surgery, nor does it appear to influence the duration of the hospital stay or the functional outcomes for patients. These findings suggest that such a treatment protocol might be considered relatively safe in terms of bleeding risk and recovery metrics for aneurysm clipping surgery. However, further prospective studies are needed to confirm these results and establish more definitive conclusions.
